# Nirsevimab Effectiveness Against Cases of Respiratory Syncytial Virus Bronchiolitis Hospitalised in Paediatric Intensive Care Units in France, September 2023–January 2024

**DOI:** 10.1111/irv.13311

**Published:** 2024-06-05

**Authors:** Juliette Paireau, Cécile Durand, Sylvain Raimbault, Joséphine Cazaubon, Guillaume Mortamet, Delphine Viriot, Christophe Milesi, Elise Daudens‐Vaysse, Dominique Ploin, Sabrina Tessier, Noémie Vanel, Jean‐Loup Chappert, Karine Levieux, Ronan Ollivier, Jamel Daoudi, Bruno Coignard, Stéphane Leteurtre, Isabelle Parent‐du‐Châtelet, Sophie Vaux

**Affiliations:** ^1^ Direction des maladies infectieuses Santé publique France Saint‐Maurice France; ^2^ Mathematical Modelling of Infectious Diseases Unit, Institut Pasteur Université Paris Cité, CNRS UMR 2000 Paris France; ^3^ Cellule Régionale Occitanie Santé publique France Toulouse France; ^4^ Service de Réanimation et Urgences Pédiatriques Hôpital Femme Mère Enfant, Hospices Civils de Lyon Bron France; ^5^ Service de soins critiques pédiatriques CHU Grenoble‐Alpes Grenoble France; ^6^ Service de Réanimation pédiatrique Hôpital Arnaud de Villeneuve Montpellier France; ^7^ Cellule Régionale Hauts‐de‐France Santé publique France Lille France; ^8^ Cellule Régionale Bourgogne‐Franche‐Comté Santé publique France Lille France; ^9^ Service de Réanimation pédiatrique CHU de La Timone Marseille France; ^10^ Cellule Régionale Occitanie Santé publique France Montpellier France; ^11^ Services d'urgences pédiatriques Centre Hospitalier Universitaire de Nantes Nantes France; ^12^ Cellule Régionale Pays de la Loire Santé publique France Nantes France; ^13^ Cellule Océan Indien Santé publique France Saint‐Denis France; ^14^ Réanimation et Soins Intensifs Pédiatriques Polyvalents, CHU Lille Université de Lille, ULR 2694 – METRICS: Évaluation des technologies de santé et des pratiques médicales Lille France

**Keywords:** bronchiolitis, case–control studies, immunisation, monoclonal antibodies, nirsevimab, paediatric intensive care units, respiratory syncytial virus, treatment effectiveness

## Abstract

In September 2023, France was one of the first countries that started a national immunisation campaign with nirsevimab, a new monoclonal antibody against respiratory syncytial virus (RSV). Using data from a network of paediatric intensive care units (PICUs), we aimed to estimate nirsevimab effectiveness against severe cases of RSV bronchiolitis in France. We conducted a case–control study based on the test‐negative design and included 288 infants reported by 20 PICUs. We estimated nirsevimab effectiveness at 75.9% (48.5–88.7) in the main analysis and 80.6% (61.6–90.3) and 80.4% (61.7–89.9) in two sensitivity analyses. These real‐world estimates confirmed the efficacy observed in clinical studies.

## 1 Introduction

Respiratory syncytial virus (RSV) infections, which mainly present in the form of bronchiolitis in children under the age of 1, are a major cause of hospitalisation in children worldwide [1]. In France, hospitalisations with RSV represent 28% of all‐cause hospitalisations in children under the age of 1 during the RSV season [2]. Seasonal RSV epidemics in France usually span from mid‐November to the end of January. After the emergence of severe acute respiratory syndrome coronavirus 2 (SARS‐CoV‐2) in 2020 and the implementation of control measures, these epidemics have experienced significant disruptions in many countries [3]. In France, the 2020–2021 season was particularly low, with a late peak, whereas the subsequent seasons of 2021–2022 and 2022–2023 showed higher impacts and earlier peaks [4, 5].

Nirsevimab, a new long‐acting anti‐RSV monoclonal antibody, has been authorised by the European Medicines Agency (EMA) in October 2022 for the prevention of RSV lower respiratory tract disease in newborns and infants during their first RSV season [6]. On 19 July 2023, the French National Authority for Health (HAS) approved the reimbursement of nirsevimab [7]. On 15 September 2023, France was one of the few European countries that started a national immunisation campaign [8, 9]. Because of the very high adherence rates in France, nirsevimab was preferentially allocated for the immunisation of newborns in maternity wards before discharge and for newborns under 1 month old in hospital wards starting from 26 September [8]. On 26 December 2023, 173,000 and 64,000 doses of nirsevimab 50 and 100 mg were distributed, respectively [10].

Nirsevimab had been shown to provide good efficacy in clinical trials [11, 12], but its effectiveness needed to be evaluated in real‐world settings. In France, in response to the increased intensity of recent RSV epidemics, bronchiolitis surveillance has been strengthened for the 2023–2024 season through a multicentric network of volunteer paediatric or neonatal intensive care units (PICUs) coordinated by Santé publique France. Most PICUs were involved in the Pediatric Intensive Care Unit Registry (PICURe). Using surveillance data from this network, we aimed to estimate nirsevimab effectiveness against severe cases of RSV bronchiolitis hospitalised in PICU in metropolitan France from 15 September 2023 to 31 January 2024.

## 2 Materials and Methods

### 2.1 Surveillance Data

PICUs participating in the multicentric surveillance network had to report the following cases to Santé publique France: infants < 2 years old presenting a severe form of bronchiolitis requiring hospitalisation in PICU, regardless of the virus causing the infection (whether identified or not). The clinical medical diagnosis of bronchiolitis was made by each clinician at the time of admission. The collected variables included age (month), sex, viral identification, administration of a preventive treatment, date and type of treatment, comorbidities (including cardiac, pulmonary, renal, liver, neuromuscular or metabolic pathologies, cancer, immunodepression and diabetes), prematurity and gestational age, date of PICU admission, respiratory support during the stay in PICU and death.

### 2.2 Study Design

We conducted a case–control study based on the test‐negative design (TND) to estimate nirsevimab effectiveness against cases of RSV bronchiolitis hospitalised in PICU.

Among all infants with bronchiolitis admitted in PICU and reported through the surveillance network (as described above), we included infants that met the following inclusion criteria: (1) admitted in PICU from 15 September 2023 to 31 January 2024, (2) in metropolitan France and (3) aged less than 1 month at the start of the study or age less than 5 months at the start of the study if they had comorbidities.

Exclusion criteria were no etiologic search, unknown preventive treatment against RSV, administration of another preventive treatment against RSV than nirsevimab (i.e. palivizumab), unknown comorbidities/prematurity or unknown sex.

In the main analysis, we excluded infants who received nirsevimab < 8 days prior to hospitalisation in PICU (taking into account RSV incubation period and time from symptom onset to PICU admission) or whose date of nirsevimab administration was unknown. In a first sensitivity analysis (SA1), infants whose date of nirsevimab administration was unknown and who were aged ≥ 1 month were included and considered as treated with nirsevimab more than 8 days before hospitalisation in PICU, because most doses were given at the maternity (before 1 month). In a second sensitivity analysis (SA2), we included as treated all infants who received nirsevimab, whatever the delay between administration of treatment and PICU admission (Table S1).

Infants were tested for RSV by multiplex PCR on nasopharyngeal swabs. Infants who tested positive for RSV were classified as cases, and those who tested negative for RSV were classified as controls.

We defined two periods based on RSV detection rates in hospital laboratories (Renal Network): (1) period of low RSV circulation from 15 September 2023 to 29 October 2023 and from 8 January 2024 to 31 January 2024 and (2) period of high RSV circulation from 30 October 2023 to 7 January 2024.

The effectiveness of nirsevimab on PICU hospitalisation for RSV bronchiolitis was estimated with a logistic regression model. The odds ratio (OR) comparing the odds of nirsevimab administration among cases to the odds among controls was adjusted for age group (0–3 months, > 3 months), sex, presence of comorbidities, prematurity and time period. Effectiveness was estimated as (1 − OR) * 100%.

## 3 Results

On 31 January 2024, 542 infants with bronchiolitis hospitalised in PICUs were reported by the surveillance network. Among them, 342 infants met the inclusion criteria for the TND study (Figure S1).

After applying the exclusion criteria, 288 infants (from 20 PICUs) were included in the main analysis, of whom 263 (91%) were aged 0–3 months and 157 (55%) were male (Table 1). RSV was identified for 238 (83%) infants (including 19 [8%] in association with another pathogen). In total, 238 cases and 50 controls were included in the main analysis. Among controls, rhinovirus was the most frequently identified pathogen (48%). Cases were younger than controls (*p* < 0.001), with a lower proportion of males (52% vs. 68%, *p* = 0.035). Over the study period, 58 (20%) infants had received nirsevimab ≥ 8 days prior to hospitalisation in PICU (mean delay: 35 days, range: 9–70). They were more likely to be premature than untreated infants (29% vs. 10%, *p* < 0.001).

**TABLE 1:** Descriptive statistics of the study population for the main analysis of nirsevimab effectiveness, France, September 2023–January 2024 (*N* = 288 infants).

Characteristic	Overall (*N* = 288)	RSV testing			Treatment by nirsevimab		
		Test negative (control) (*N* = 50)	Test positive (case) (*N* = 238)	*p* ^a^	Untreated (*N* = 230)	Treated (*N* = 58)	*p* ^a^
	*n* (%)^b^	*n* (%)^b^	*n* (%)^b^		*n* (%)^b^	*n* (%)^b^	
Age group (months)				< 0.001			0.3
0–3	263 (91%)	38 (76%)	225 (95%)		208 (90%)	55 (95%)	
4–8	25 (9%)	12 (24%)	13 (5%)		22 (10%)	3 (5%)	
Sex				0.035			0.3
Female	131 (45%)	16 (32%)	115 (48%)		108 (47%)	23 (40%)	
Male	157 (55%)	34 (68%)	123 (52%)		122 (53%)	35 (60%)	
Viral identification^c^							
RSV	238 (83%)	0 (0%)	238 (100%)	< 0.001	201 (87%)	37 (64%)	< 0.001
Rhinovirus	42 (15%)	24 (48%)	18 (8%)	< 0.001	26 (11%)	16 (28%)	0.002
Metapneumovirus	6 (2%)	5 (10%)	1 (0%)	< 0.001	3 (1%)	3 (5%)	0.10
Other virus	23 (8%)	23 (46%)	0 (0%)	< 0.001	14 (6%)	9 (16%)	0.028
Not identified	12 (4%)	12 (24%)	0 (0%)	< 0.001	8 (3%)	4 (7%)	0.3
Period of PICU admission				0.007			0.8
15 September 2023–29 October 2023	30 (10%)	10 (20%)	20 (8%)		24 (10%)	6 (10%)	
30 October 2023–7 January 2024	251 (87%)	37 (74%)	214 (90%)		201 (87%)	50 (86%)	
8–31 January 2024	7 (2%)	3 (6%)	4 (2%)		5 (2%)	2 (3%)	
Comorbidities^d^				0.014			0.2
No	254 (88%)	39 (78%)	215 (90%)		206 (90%)	48 (83%)	
Yes	34 (12%)	11 (22%)	23 (10%)		24 (10%)	10 (17%)	
Prematurity				< 0.001			< 0.001
No	249 (86%)	34 (68%)	215 (90%)		208 (90%)	41 (71%)	
Yes	39 (14%)	16 (32%)	23 (10%)		22 (10%)	17 (29%)	
If prematurity, gestational age (mean [SD], week)	33 (4)	32 (5)	34 (2)	0.7	34 (3)	32 (4)	0.8
Nirsevimab				< 0.001			< 0.001
No	230 (80%)	29 (58%)	201 (84%)		230 (100%)	0 (0%)	
Yes	58 (20%)	21 (42%)	37 (16%)		0 (0%)	58 (100%)	
Respiratory support				> 0.9			0.6
Non‐invasive ventilation	209 (73%)	36 (72%)	173 (73%)		165 (72%)	44 (76%)	
High‐flow nasal cannula	57 (20%)	10 (20%)	47 (20%)		48 (21%)	9 (16%)	
Invasive ventilation	18 (6%)	3 (6%)	15 (6%)		13 (6%)	5 (9%)	
Extracorporeal assistance	0 (0%)	0 (0%)	0 (0%)		0 (0%)	0 (0%)	
None/not specified	4 (1%)	1 (2%)	3 (1%)		4 (2%)	0 (0%)	
Death	0 (0%)	0 (0%)	0 (0%)	NA	0 (0%)	0 (0%)	NA

^a^ *p* values from Pearson's chi‐squared test and Fisher exact test for categorical variables and Wilcoxon rank sum test for continuous variables.

^b^ *n* (%): percentages may not total 100 due to rounding.

^c^ Percentages do not total 100 due to co‐infections by several pathogens.

^d^ Include cardiac, pulmonary, renal, liver, neuromuscular or metabolic pathologies, cancer, immunodepression and diabetes.

In the main analysis, we estimated that adjusted nirsevimab effectiveness against cases of RSV bronchiolitis hospitalised in PICU was 75.9% (95% confidence interval [CI] 48.5‐88.7). In the sensitivity analyses, nirsevimab effectiveness was estimated at 80.6% (61.6–90.3) for SA1 (312 infants included) and 80.4% (61.7–89.9) for SA2 (319 infants included) (Table 2).

**TABLE 2:** Estimated effectiveness of nirsevimab against cases of RSV bronchiolitis hospitalised in PICU, France, September 2023–January 2024.

Analysis	Controls not treated by nirsevimab	Controls treated by nirsevimab	Cases not treated by nirsevimab	Cases treated by nirsevimab	Unadjusted effectiveness (95% CI)	Adjusted effectiveness^a^ (95% CI)
Main analysis (*N* = 288)	29	21	201	37	74.4% (50.5–86.8)	75.9% (48.5–88.7)
Sensitivity analysis 1 (*N* = 312)	29	35	201	47	80.5% (65.0–89.1)	80.6% (61.6–90.3)
Sensitivity analysis 2 (*N* = 319)	29	38	201	51	80.5% (65.4–89.0)	80.4% (61.7–89.9)

^a^ Adjusted for age group (0–3 months, 4–8 months), sex, presence of comorbidities, prematurity and time period (period of low RSV circulation or period of high RSV circulation).

## 4 Discussion

Our results point towards a high effectiveness of nirsevimab in preventing severe RSV bronchiolitis in infants requiring PICU admission from 75.9% (48.5–88.7) to 80.6% (61.6–90.3) depending on the assumptions. These real‐world estimates are in line with the efficacy observed in clinical studies: the efficacy of nirsevimab was evaluated in clinical trials against very severe medically attended RSV lower respiratory tract disease between 64.2% (−12.1–88.6) and 87.5% (62.9–95.8) depending on gestational age [6, 11] and at 75.7% (32.8–92.9) in a pragmatic trial [12]. This strong effectiveness is also consistent with a Spanish study looking at RSV‐related hospitalisations [13] and French surveillance data showing a reduced impact of the 2023–2024 bronchiolitis epidemic on hospitalisations of infants under 3 months compared to previous years and in contrast to older infants [14].

To our knowledge, this study is the first to provide real‐world estimates of nirsevimab effectiveness against severe cases of RSV bronchiolitis hospitalised in PICU. The strength of our study is the use of the TND, which allows timely estimation of effectiveness based on relatively limited surveillance data and reduces confounding bias due to differences in behaviour and access to care between cases and controls. Among the 41 PICUs in France, a large proportion (49%) took part in the TND study, which should ensure that our results are generalisable to the French population. Bias may still exist if healthcare use differs between infants treated or not with nirsevimab with relatively less severe or more severe illness [15]. However, this bias appears very limited in an analysis focusing only on severe cases admitted to PICU. The main limitation of our study is the small sample size, especially for the controls, which does not allow for subgroup analyses or matching of cases and controls. In addition, bias may exist if the preventive treatment and administration dates were less frequently reported when the pathogen was not RSV, leading to an underestimation of effectiveness. We attempted to mitigate this potential bias through sensitivity analyses. Other potential biases could be related to outcome misclassification due to the performance of diagnostic assays, which could vary from one hospital to another. However, we believe that these potential biases are limited by the fact that cases and controls were recruited from the same PICUs and therefore tested using the same tests within each hospital.

Nirsevimab seems to be able to occupy a significant position in the therapeutic arsenal for preventing RSV infections, although it must be compared to other preventive treatments in development. The RSV vaccine for pregnant women is actually under evaluation by health authorities and not yet available in France.

## Author Contributions

**Juliette Paireau:** conceptualization, methodology, data curation, formal analysis, writing–original draft, writing–review and editing. **Cécile Durand:** data curation, investigation, writing–review and editing. **Sylvain Raimbault:** data curation, writing–review and editing. **Joséphine Cazaubon:** data curation, writing–review and editing. **Guillaume Mortamet:** data curation, investigation, writing–review and editing. **Delphine Viriot:** investigation, writing–review and editing. **Christophe Milesi:** data curation, investigation, writing–review and editing. **Elise Daudens‐Vaysse:** data curation, investigation, writing–review and editing. **Dominique Ploin:** data curation, investigation, writing–review and editing. **Sabrina Tessier:** data curation, investigation, writing–review and editing. **Noémie Vanel:** data curation, investigation, writing–review and editing. **Jean‐Loup Chappert:** data curation, investigation, writing–review and editing. **Karine Levieux:** investigation, writing–review and editing. **Ronan Ollivier:** data curation, investigation, writing–review and editing. **Jamel Daoudi:** investigation, writing–review and editing. **Bruno Coignard:** investigation, writing–review and editing. **Stéphane Leteurtre:** data curation, investigation, writing–review and editing. **Isabelle Parent‐du‐Châtelet:** conceptualization, methodology, investigation, writing–review and editing. **Sophie Vaux:** conceptualization, methodology, data curation, investigation, writing–original draft, writing–review and editing.

## Ethics Statement

The protocol was conducted in agreement with the Helsinki declaration. We obtained authorisation from the French Data Protection Agency (registration numbers 1929497 and 2224869).

## Consent

The study was considered as non‐interventional research according to article L1221‐1.1 of the public health code in France and only requires the non‐opposition of the patient (per article L1211‐2 of the public health code). All data were anonymised before use.

## Conflicts of Interest

The authors declare no conflicts of interest.

## Supporting information


**Figure S1.** Flowchart of study inclusion and exclusion criteria.
**Table S1.** Definition of the main analysis and the two sensitivity analyses (SA1 and SA2).

## Data Availability

The data that support the findings of this study are available from the corresponding author upon reasonable request.
