# Supplementary material for: Nirsevimab Effectiveness Against Cases of Respiratory Syncytial Virus Bronchiolitis Hospitalised in Paediatric Intensive Care Units in France, September 2023–January 2024
Source: Influenza Other Respir Viruses. 2024 Jun 5;18(6):e13311. doi: 10.1111/irv.13311 (PMC11154801; doi:10.1111/irv.13311)
Supplement: Supplementary file 1 — Figure S1. Flowchart of study inclusion and exclusion criteria. Table S1. Definition of the main analysis and the two sensitivity analyses (SA1 and SA2). [file IRV-18-e13311-s001.docx]

**Supplementary material**

**Nirsevimab effectiveness against cases of respiratory syncytial virus bronchiolitis hospitalised in pediatric intensive care units in France, September 2023 - January 2024**

Juliette Paireau, Cécile Durand, Sylvain Raimbault, Joséphine Cazaubon, Guillaume Mortamet, Delphine Viriot, Christophe Milesi, Elise Daudens-Vaysse, Dominique Ploin, Sabrina Tessier, Noémie Vanel, Jean-Loup Chappert, Karine Levieux, Ronan Ollivier, Jamel Daoudi, Bruno Coignard, Stéphane Leteurtre, Isabelle Parent-du-Châtelet, Sophie Vaux

**Figure S1. Flowchart of study inclusion and exclusion criteria.**


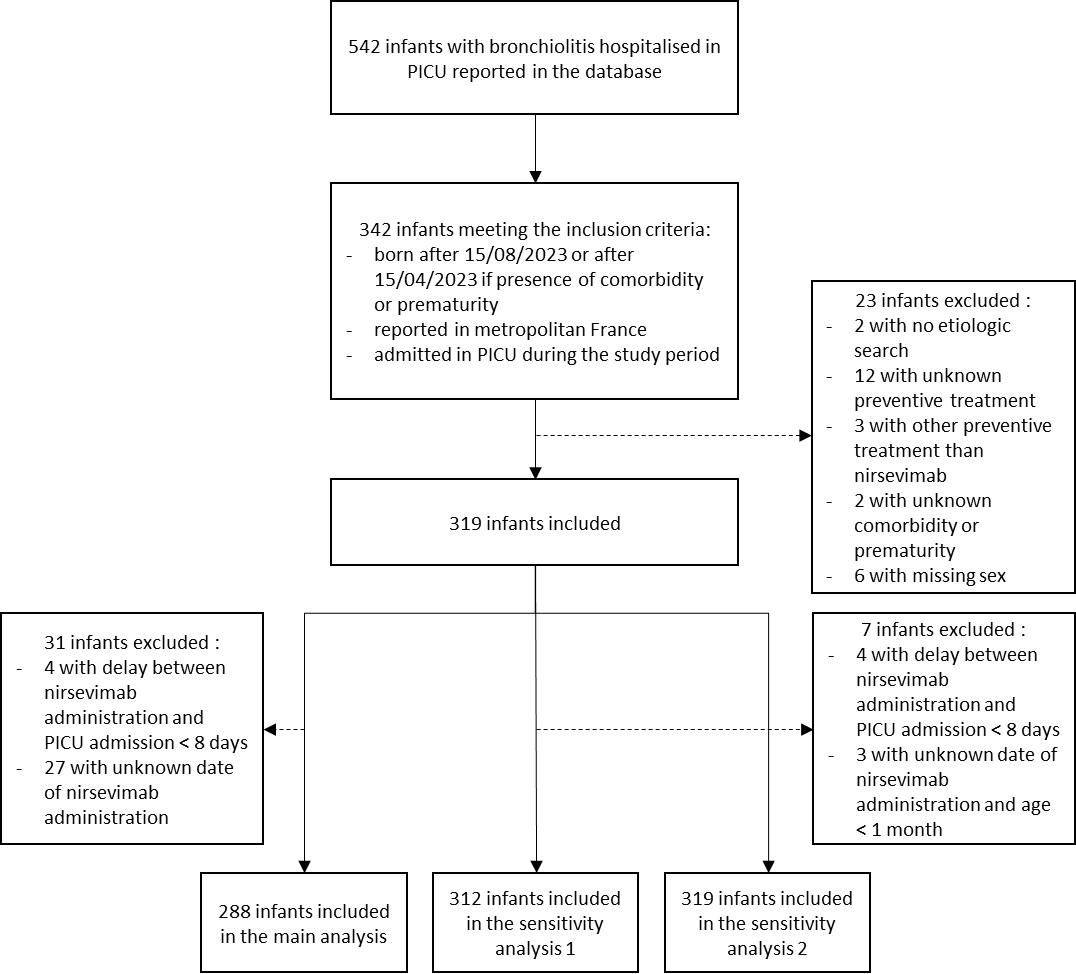


**Table S1. Definition of the main analysis and the two sensitivity analyses (SA1 and SA2).** The three analyses differ in the way they include/exclude infants who received nirsevimab based on the age and the time T between nirsevimab administration and PICU admission.

| **Analysis** | **Definition** | | | | |
| --- | --- | --- | --- | --- | --- |
|  | **Did not receive nirsevimab** | **Received nirsevimab** | | | |
|  |  | **T ≥ 8 days** | **T < 8 days** | **Unknown date of nirsevimab administration and age = 0 month** | **Unknown date of nirsevimab administration and age ≥ 1 month** |
| Main | Included as “untreated” | Included as “treated” | Excluded | Excluded | Excluded |
| SA1 |  |  | Excluded | Excluded | Included as “treated” |
| SA2 |  |  | Included as “treated” | Included as “treated” | Included as “treated” |
